# Longitudinal analysis of gamma delta T cell subsets during malaria infections in Malian adults

**DOI:** 10.1186/s12936-019-2702-5

**Published:** 2019-03-12

**Authors:** Hama Diallo, Abdoulaye Katile, Jennifer L. Kwan, Mahamadou S. Sissoko, Sara A. Healy, Ogobara K. Doumbo, Patrick E. Duffy, Irfan Zaidi

**Affiliations:** 1 Malaria Research Training Center, International Center of Excellence in Research, Faculty of Medicine and Pharmacy of the University of Sciences, Techniques and Technologies of Bamako, Bamako, Mali; 2 Laboratory for Malaria Immunology and Vaccinology, National Institute of Allergy and Infectious Diseases, National Institutes of Health, Rockville, MD USA

**Keywords:** Clinical malaria, *Plasmodium falciparum*, γδ T cells, Longitudinal analysis

## Abstract

**Background:**

Immunity that limits malarial disease is acquired over time, but adults living in endemic areas continue to become infected and can require treatment for clinical illness. Gamma delta (γδ) T cells, particularly the Vδ2+ subset, have been associated with development of clinical malaria in children. In this study, the dynamics of total γδ T cells, Vδ2+ and Vδ2− T cells were measured during a malaria transmission season in Malian adults.

**Methods:**

This study explored γδ T cell dynamics and *Plasmodium falciparum* infection outcomes over the course of the malaria transmission season in Malian adults enrolled in the placebo arm of a double-blind randomized vaccine trial. All volunteers were treated with anti-malarial drugs prior to the start of the transmission season and blood smears were assessed for *P. falciparum* infection every 2 weeks from July 2014 to January 2015. The study participants were stratified as either asymptomatic infections or clinical malaria cases. Vδ2+ and Vδ2− γδ T cell frequencies and activation (as measured by CD38 expression) were measured in all study participants at baseline and then every 2 months using a whole blood flow cytometry assay.

**Results:**

Forty of the forty-three subjects became infected with *P. falciparum* and, of those, 21 individuals were diagnosed with clinical malaria at least once during the season. The γδ T cell percentage and activation increased over the duration of the transmission season. Both the Vδ2+ and Vδ2− γδ T cells were activated by *P. falciparum* infection.

**Conclusion:**

γδ T cells increased during a malaria transmission season and this expansion was noted in both the Vδ2+ and Vδ2− γδ T cells. However, neither expansion or activation of either γδ T cell subsets discriminated study participants that had asymptomatic infections from those that had clinical malaria cases.

## Background

Malaria remains an acute public health problem with 216 million reported cases that led to 445,000 deaths in 2016. Sub-Saharan Africa continues to be the most affected region and accounted for 91% of malaria cases and 90% of malaria deaths in 2016 [1]. The most severe form of malaria is caused by *Plasmodium falciparum* and accounts for the majority of deaths, especially in children under the age of 5 years.

Despite repeated infections, sterile immunity to *P. falciparum* infection is not thought to develop [2, 3]. However, there is evidence for clinical immunity against malaria as evidenced by reduced clinical cases and greater proportions of asymptomatic infections in older children and adults [4]. Development of clinical immunity has been associated with blunted Th1 immune responses, and increases in immunoregulatory mechanism, such as T regulatory cells and IL-10 production from CD4 T cells [5–7]. More recently, reduced responses of gamma delta (γδ) T cell responses to *P. falciparum* infections were associated with development of clinical immunity and this was attributed to the Vδ2 γδ T cell subset in particular [8]. γδ^+^ T lymphocytes have been reported to be elevated in peripheral blood [9] and spleens of individuals with acute or convalescent *P. falciparum* infection [10]. The high level of these cells has also been observed during febrile paroxysms of *Plasmodium vivax* infection [11]. Proportional expansion of both Vδ1^+^ and Vδ2^+^ subsets has been reported in acute *P. falciparum* infection [12, 13].

γδ T cells are a specialized subset that have features of both innate and adaptive immune cells [14]. Their T cell receptor consists of a gamma and a delta chain and are usually identified according to the delta chain expressed on the surface. Hence, γδ T cells are classified as Vδ1+, Vδ2+ and a minor subset, Vδ3+. The Vδ2+ γδ T cells are typically the predominant circulating subset in the blood and their TCR consists of a Vδ2 and a Vγ9 chain [15]. The Vδ1+ T cell subset can constitute the major subset of γδ T cells but they have a wider gamma chain usage including Vγ9 [16].

The PfSPZ vaccine trial in Mali evaluated the efficacy of the Sanaria^®^ whole sporozoite vaccine to *P. falciparum* infection in the field and required very intense follow-up of study participants [17]. Using a treatment-reinfection approach, the clinical outcomes of *P. falciparum* infection were evaluated in the study participants. Furthermore, whether the dynamics of γδ T cells would distinguish asymptomatic infections and clinical malaria cases was evaluated. In over half of the adults in the study, *P. falciparum* infections resulted in clinical malaria cases, but neither the percentage nor activation of γδ T cells discriminated clinical malaria cases from asymptomatic infections.

## Methods

### Human ethics statement

All subjects provided written informed consent before screening. Ethics Committee (EC) of Faculté de Médecine de Pharmacie et d’OdontoStomatologie (FMPOS) and the NIAID Institutional Review Board (IRB) approved the study protocol (Number: 14-I-N010).

### Study design

The data from this study were from the placebo group in the recently concluded PfSPZ Vaccine trial. This study was conducted in Mali through the Malaria Research Training Center (MRTC) of the Medical School of the University of Sciences, Techniques and Technologies of Bamako (USTTB) from August 2014 to January 2015. The study was conducted according to Good Clinical Practices including external monitoring of the data.

### Study participants

Forty-four of the 48 subjects that were enrolled in the placebo group completed the study and these were used for this analysis. The volunteers were healthy adults between 18 and 35 years old and were screened for eligibility based on residency in the village, their medical and family history, as well as their physical examination. None of the female volunteers were pregnant or lactating. Serology for HIV, hepatitis B and C was negative for all volunteers before inclusion. All volunteers provided written informed consent before screening.

### Study procedures

Study participants were assayed at the start of the transmission season (day/week 0), 6 weeks (day 38) and 14 weeks (day 98) later, and at the end of the study at 22 weeks (day 154) as malaria transmission waned. At each visit, 1 ml of whole blood was drawn for parasite detection by thick smear and immunological ex vivo assays. Volunteers were treated with artemether/lumefantrine approximately 4 weeks before the day 0 visit. Participants were encouraged to make unscheduled visits anytime during the study period if they experienced any clinical symptoms.

### Peripheral blood smears

Thick blood smears were prepared from finger prick samples starting at day 0 and then every 2 weeks until the end of the study. Certified technicians examined the smears following established procedures for malaria slide reading within the MRTC, Mali. Blood films were considered positive if at least two unambiguous *Plasmodium* parasites were seen.

### Clinical malaria and asymptomatic infection case definitions

In accordance with Malian national treatment guidelines, symptomatic malaria was defined as *P. falciparum* asexual parasitaemia accompanied by an axillary temperature of at least 37·5 °C and/or clinical signs and symptoms compatible with malaria. If a subject was diagnosed with symptomatic malaria, subjects were treated with artemether-lumefantrine.

Clinical malaria cases were defined as having an axillary temperature of 37.5 °C, clinical signs and symptoms of malaria. All per protocol (scheduled) anti-malarial treatments were administered by directly observed treatment by study staff. Participants were instructed that if they vomited within 1 h of taking the drug, they should return to the clinical trial centre to receive a repeat dose. Asymptomatic *P. falciparum* infection cases were not treated, as per national malaria guidelines.

### Immunological analysis

One ml of whole blood was collected in a sodium heparin tube from each volunteer at study day 0, day 38, day 98 and day 154 during the period of the study, which coincided with a clinical follow-up visit day. One hundred fifty μl of whole blood was used for staining with a cocktail of antibodies against: anti-CD3 BV650, anti-CD8 APC-H7, anti-γδTCR PE, anti-Vδ2 FITC purchased from e-bioscience. Red blood cells were lysed using the BD FACS Lyse solution and the cells were washed two times with PBS and acquired on a LSRII flow cytometer equipped with a Blue (488 nm), Red (633 nm) and Violet laser (405 nm). γδT cells (Vδ2+ and Vδ2− populations) were enumerated as a percentage of total CD3 T cells.

### RNA sequencing

Whole blood was collected in PAXgene tubes from study subjects at the begininning of the transmission season (Day 0). The methods used for RNA isolation, sequencing and subsequent bioinformatics analysis are previously described [18]. The raw data was processed as previously described and the Reads Per Kilobase of transcript per Million mapped reads (RPKM) were generated for all genes. Only T cell receptor gamma and delta genes were selected for further analysis. The relative expression (as assessed by mean RPKM values) of T cell receptor V delta (TRDV1, TRDV2 and TRDV3) and gamma genes (TRGV1, TRGV2, TRGV3, TRGV5, TRGV5P, TRGV6, TRGV7, TRGV8, TRGV9, TRGV10 and TRGV11) were compared.

### Statistical analyses

Statistical analyses were performed using Prism7 (Graph Pad). The associations between immunologic outcomes with exposure variables were examined, including prior cumulative incidence, concurrent parasitemia at the time of the assay, and anti-malarial treatment allocation. The comparisons of cellular percentages were performed using the nonparametric Kruskal–Wallis to compare all the groups and Mann–Whitney test to compare between the groups. A p value of < 0.05 was considered statistically significant.

## Results

### *Plassmodium falciparum* infections

Malaria transmission in Mali is highly seasonal and closely aligned to the rainy season that begins in July and concludes in December. The study was conducted in Donéguébougou, Mali which is 30 km from the capital Bamako. Forty-four subjects that were enrolled in the placebo arm of the PfSPZ Vaccine trial in Mali were used for the analysis of γδ T cells across the malaria transmission season in 2014.

**Fig. 1:**
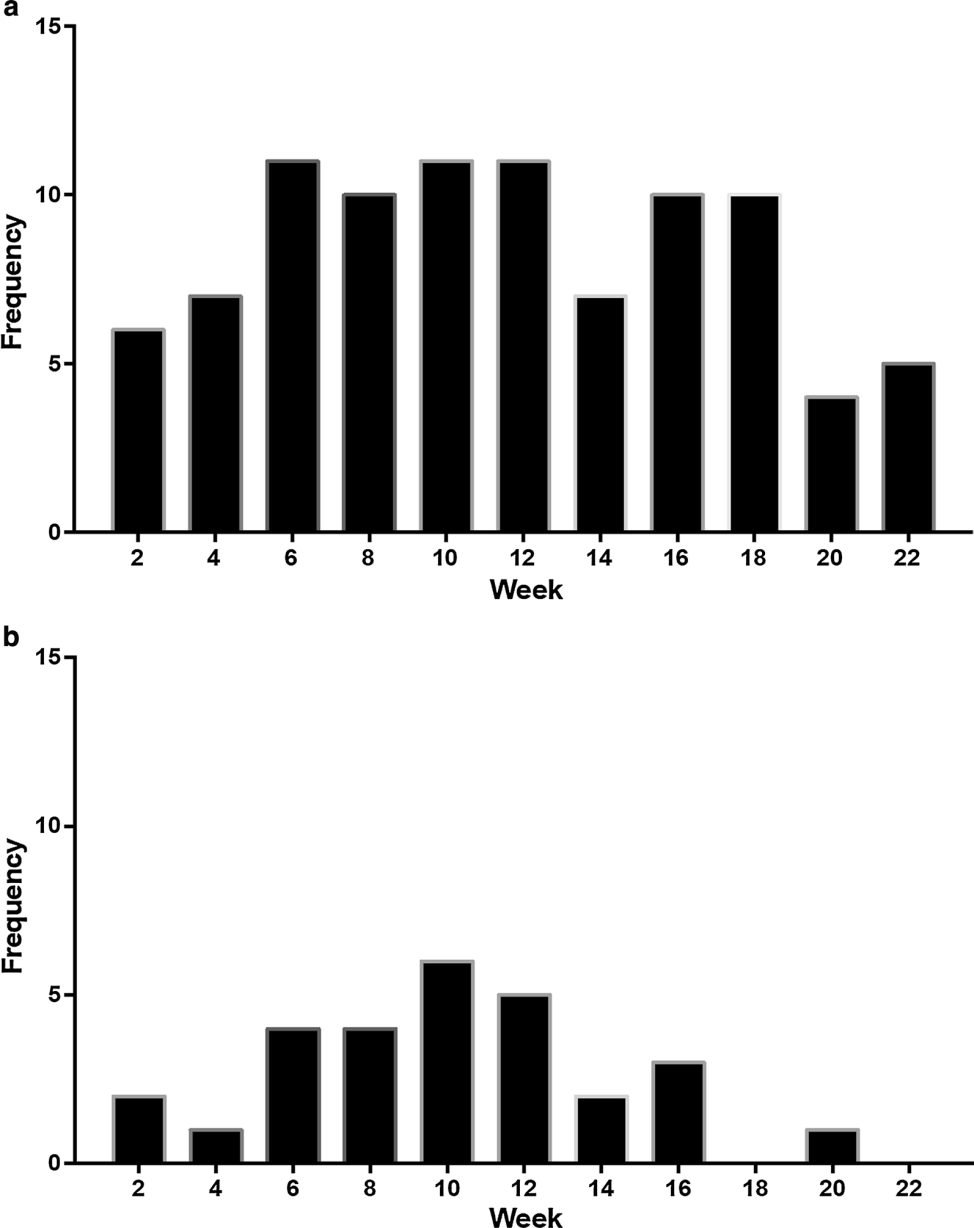
Frequency of *Plasmodium falciparum* infections. **a** Number of positive blood smears recorded bi-weekly. **b** Number of clinical malaria cases recorded bi-weekly

The volunteers had a median age of 24 years (range 18–35) and were predominantly male (41/44). A total of 119 positive blood smears were recorded in the 41 participants. Consecutive positive blood smears recorded within 28 days in individual subjects were counted as a single infection. Three study volunteers remained uninfected throughout the study period, as assessed by blood smear results, and were excluded from further analysis. Additional details of study participants were reported in the publication describing the PfSPZ Vaccine trial results in Mali [17]. The frequency of positive blood smears was highest during weeks 8–12, with 15 recorded infections, and transmission continued till the end of the follow-up period at week 22. Study participants were classified as either clinical malaria or asymptomatic infection groups. Any volunteer who was treated for symptomatic malaria anytime during the season was included in the clinical malaria group. Volunteers who had a positive blood smear but had no symptoms were included in the asymptomatic infection group. A total of 28 clinical malaria cases were recorded in 21 unique individuals during the transmission season. Clinical malaria cases peaked between weeks 10 and 12 (Fig. 1a, b).

**Fig. 2:**
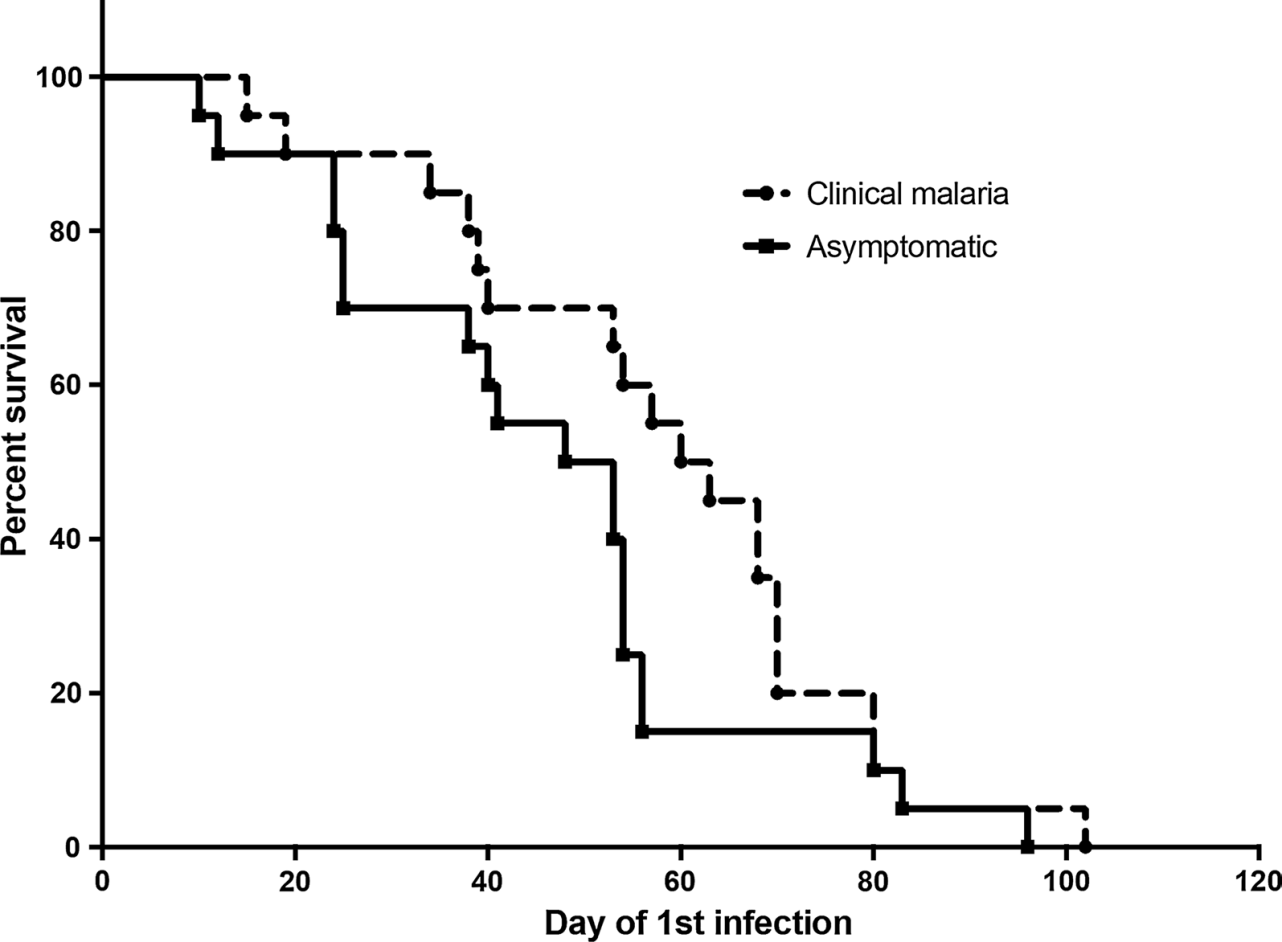
Time to first infection between clinical cases and asymptomatic infections

The time to first infection between the clinical and asymptomatic groups was analyzed and median time to first infection was not significantly different between the two groups (61.5 versus 50 days, p = 0.1238, Log-Rank test, Fig. 2).

### γδ T cell frequencies

**Fig. 3:**
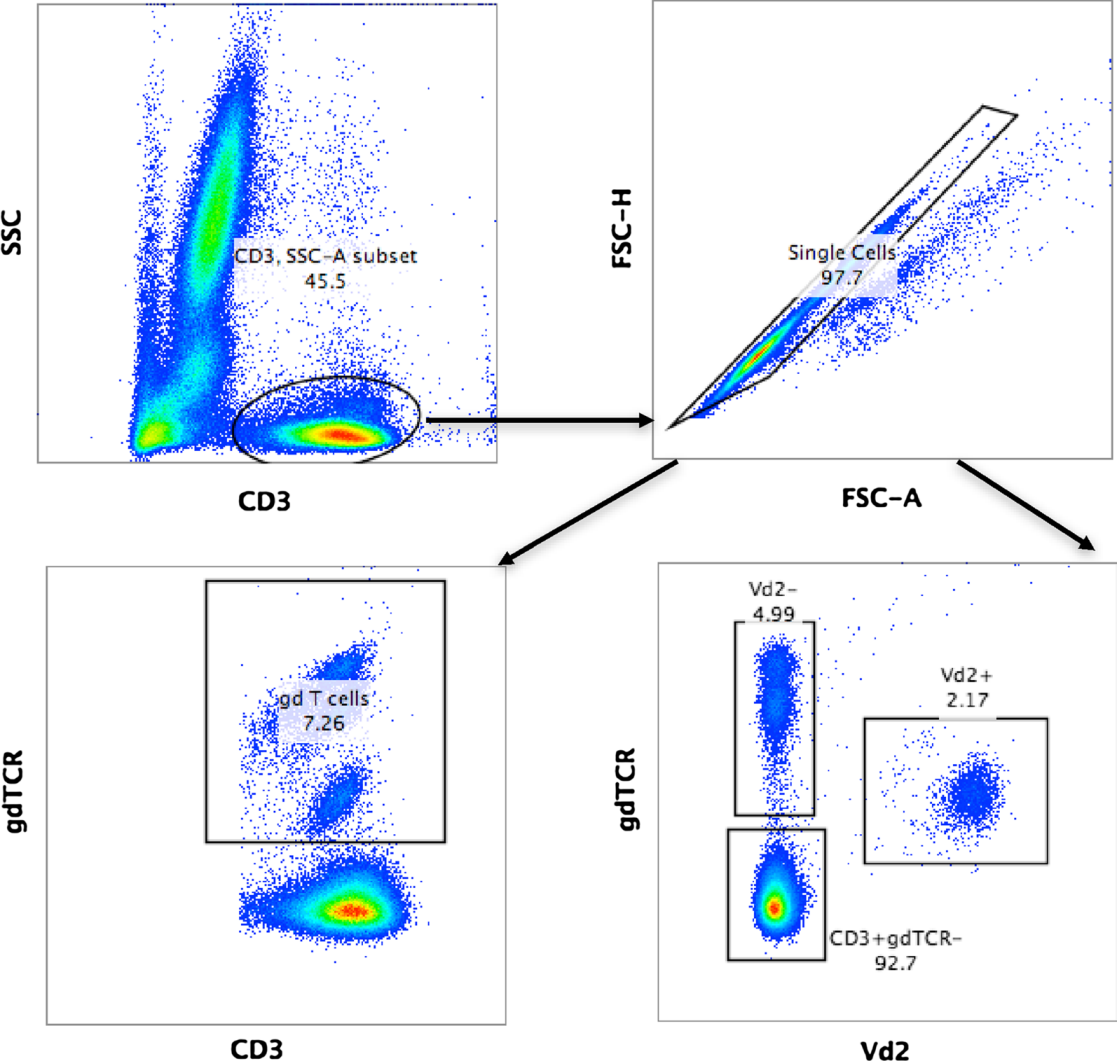
Flow cytometry gating strategy. Total CD3 + events were gated and then doublets were excluded. Total γδTCR + T cells, Vδ2+ and Vδ2− γδTCR + T cells were then enumerated as a percentage of CD3+ events

During the course of the study, whole blood was used to enumerate total γδ T cells and the Vδ2+γδ T cell subset. The gating strategy is shown in Fig. 3. At least two distinct populations of γδ T cells were visible and the Vδ2 + subset was distinguished by lower expression levels of γδ TCR as previously shown [18]. CD45RO expression was only seen on the Vδ2+ subset while a significant proportion of Vδ2− γδ T cells also expressed CD8α.

### V gamma and delta chain usage

**Fig. 4:**
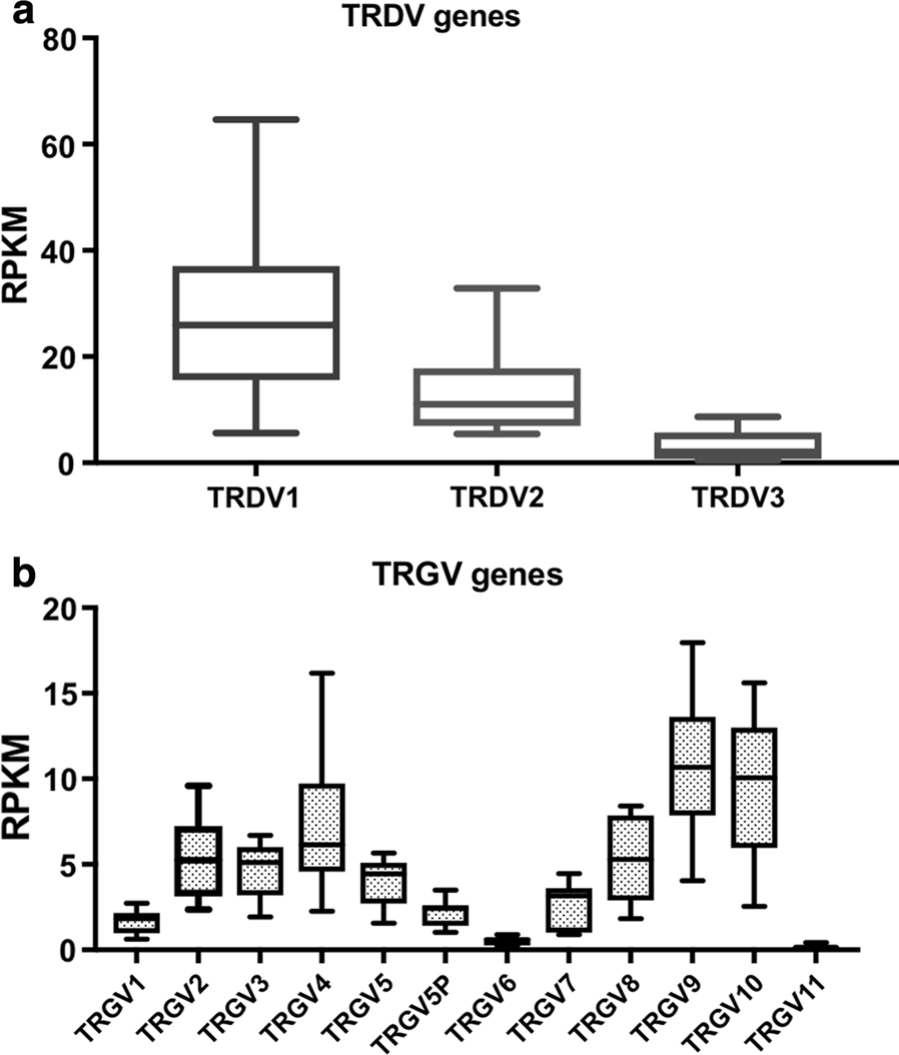
V delta and gamma gene expression. Transcriptomic analysis of RPKM of **a** V delta genes, **b** V gamma genes in 6 study volunteers

RNA from 6 subjects used in this analysis were previously used for sequencing and the results were submitted to a publically available repository [18]; (Accession number: GSE86308). The data were used to infer the relative abundance of γδ T cell subsets. Two subjects were in the clinical malaria group and 4 in the asymptomatically infected group. Comparison of the RPKM values of the TRVD genes showed that overall the expression of TRDV1 was highest followed by TRDV2 and lastly TRDV3 (Fig. 4a). A similar analysis was done for the TRGV genes. TRGV9 had the highest RPKM values followed by TRGV10 (Fig. 4b).

### Comparison of γδ T cell frequencies

**Fig. 5:**
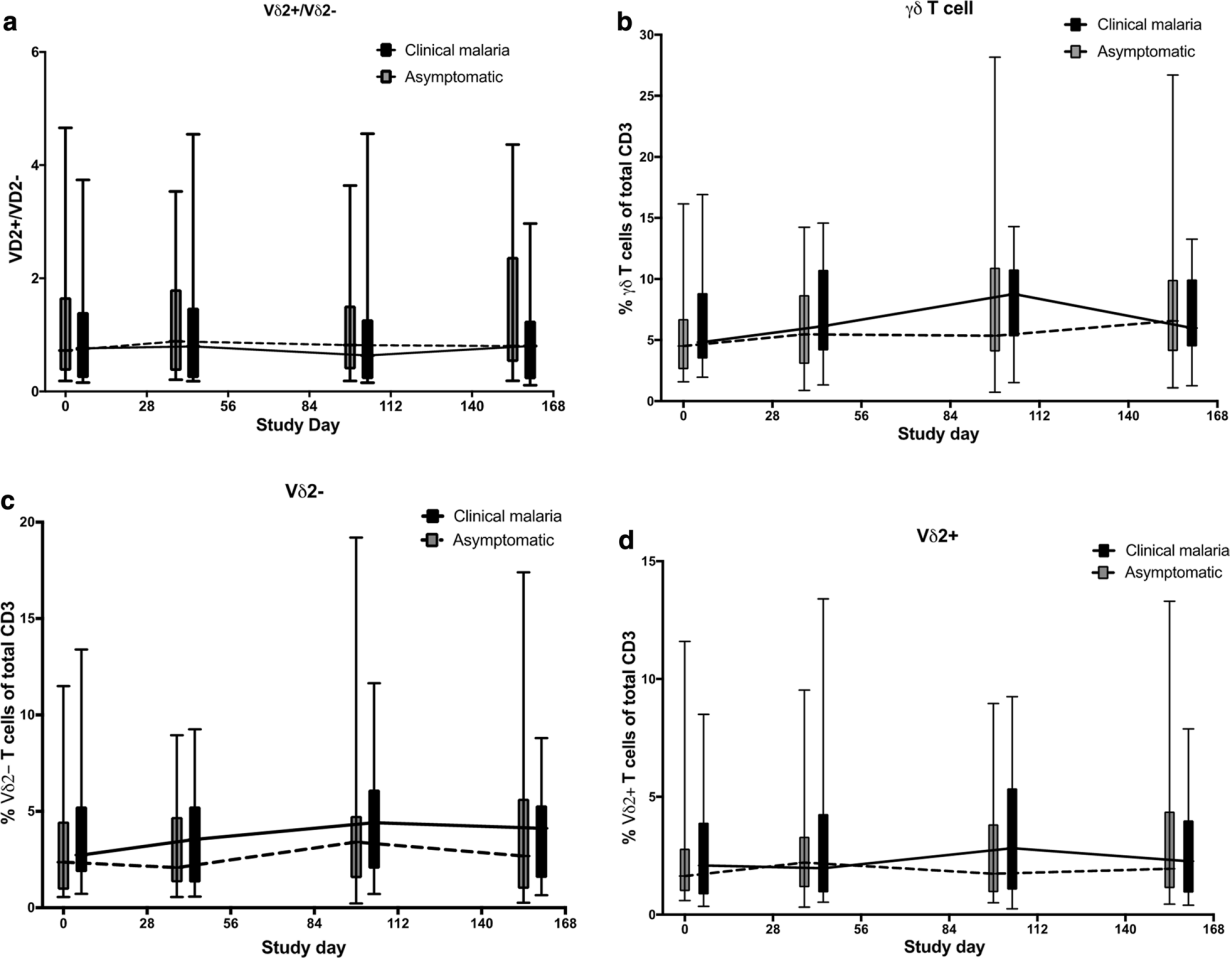
Gamma delta T cells during the malaria transmission season. Comparison of the **a** ratio of Vδ2+ to non-Vδ2 γδ T cells, **b** total γδ T cells **c** Vδ2− γδ T cell subset and **d** Vδ2+ γδ T cells between clinical malaria cases versus asymptomatic infections. The data are represented as Box and Whisker plots of medians with ranges. The trend lines indicate the medians for each group

The levels of total γδ T cells, Vδ2 + and Vδ2- subsets were compared between clinical malaria cases and asymptomatic infections at the start of the transmission season (day 0), 6 weeks (day 38), 14 weeks (day 98) and at the end of the study at 22 weeks (day 154). The ratio of Vδ2+ to Vδ2− γδ T cells was similar at all time points between volunteers who had asymptomatic or clinical malaria cases (Fig. 5a). Comparison of the total percentage of γδ T cell in all study subjects showed an increase from baseline and peaked at day 98 in all study subjects but the differences were not statistically significant at any time point. Comparison of the Vδ2− and Vδ2+ subsets dynamics did not discriminate individuals who developed clinical malaria versus only asymptomatic infections during the season (Fig. 5c, d).

### γδ T cell activation

**Fig. 6:**
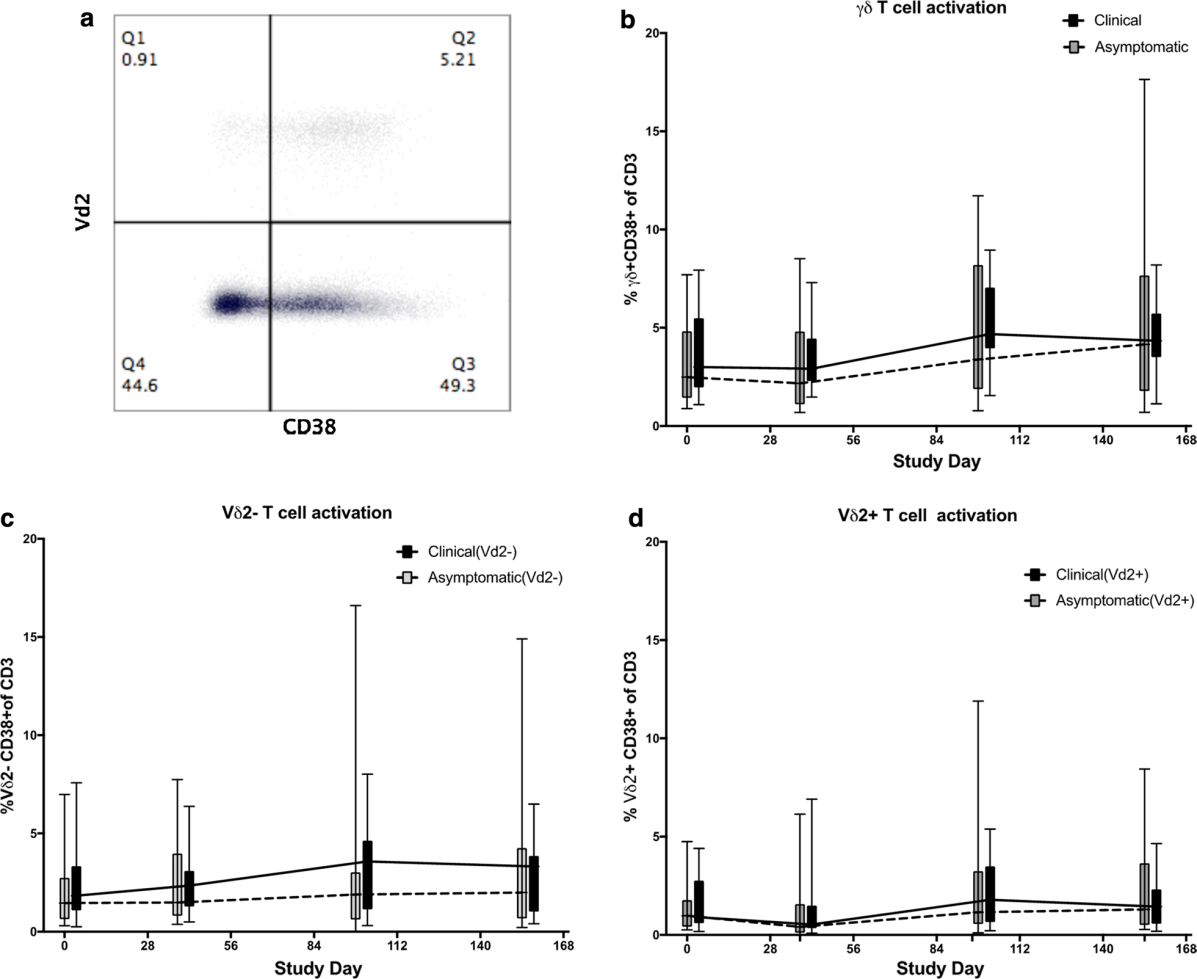
Gamma delta T cell activation during the malaria transmission season. **a** Representative flow cytometry plot of CD38 expression in Vδ2+ and Vδ2− γδ T cells. **b** Comparison of the total γδTCR + CD38 + T cells. **c** Vδ2− γδTCR + CD38 + and **d** Vδ2+ γδ T cells between clinical malaria cases versus asymptomatic infections. All values are expressed as percentage of total CD3 T cells. The data are represented as Box and Whisker plots of medians with ranges. The trend lines indicate the medians for each group

Activation of γδ T cells was assessed by measuring CD38 co-expression (Fig. 6a). The percent of γδTCR + CD38 + from all subjects increased from baseline to the end of the study at D154 (Fig. 6a, p = 0.01, paired t test). The next analysis evaluated the activation levels within the Vδ2− and Vδ2+ γδ T cells respectively. The percentage of Vδ2−CD38 + and Vδ2+ CD38 + γδ T cells was significantly higher at day 154 than at baseline (Fig. 6c, p = 0.037 and Fig. 6d, p = 0.0036, respectively, paired *t* test). The percentages of total γδ T cells that were CD38 + , Vδ2−CD38 + or Vδ2+ CD38 + were not significantly different between the clinical malaria and asymptomatically infected groups.

## Discussion

In the current study, malaria infections were monitored in Malian adults who were enrolled in the placebo arm of a recently concluded vaccine trial [17] and compared the levels, activation and subsets of γδ T cell prior to and during the transmission season. This study followed a treatment-reinfection design and this allowed us to monitor the clinical outcome of infection and relate this to changes in γδ T cell dynamics. Almost all (41/44, 93%) of participants were infected at least once, confirming the absence of sterilizing immunity as has been previously documented in semi-immune populations [3]. However, infection outcomes differed where half the participants developed clinical malaria at least once and required treatment, whereas others only experienced asymptomatic infection and displayed no signs of disease. There was no significant difference in the time to first infection between the clinical malaria and asymptomatic infections, suggesting that immune factors are contributing to the differential outcome instead of differences in infecting *P. falciparum* parasites.

Recent studies have highlighted a potential role for γδ T cells in malaria disease pathogenesis [8, 19]. During this study γδ T cells in adults were measured to evaluate whether the phenotype, frequency and activation were altered during the malaria transmission season and whether they associated with risk of malarial disease. The profile of γδ T cells was dominated by Vδ2− γδ T cells, which were higher than Vδ2+ γδ T cells in the majority of the volunteers in this study. This is in stark contrast to populations living in developed countries, where the predominant circulating γδ T cell subsets are the Vδ2+T cells [15]. Previous studies [20, 21] have demonstrated that adults living in malaria endemic regions have greater proportions of Vδ1+γδ T cells in the periphery when compared to malaria naïve individuals. While we did not stain for Vδ1+γδ T cells, the transcriptomics analysis performed from a subset of volunteers in this study clearly demonstrated that the expression profile of the V delta genes was dominated by TRDV1 gene, which codes for the V delta 1 chain. This may have important implications for malaria vaccines (especially whole organism vaccines). It has been shown that Vδ2+, but not Vδ1, γδ T cells are expanded after administration of the whole organism PfSPZ Vaccine in Malian adults [18]. While it is clear that Vδ2+ γδ T cells respond to metabolites of the *Plasmodium* DOXP pathway [22], it remains unclear how Vδ1+ T cells are activated by *P. falciparum* infections and this requires further exploration. Furthermore, determining whether the difference in γδ T cell profiles between malaria naïve and malaria exposed populations is due to host genetics or pathogen exposure warrants closer examination.

Recent studies have highlighted the role of γδ T cell levels in the pathogenesis of malaria in children [8, 19, 23]. These studies collectively suggest that Vδ2+T cell activation is associated with onset of clinical malaria and their responses are quickly blunted as a result of increased expression of inhibitory molecules such as TIM-3 [24]. In this study, the percentage of Vδ2+γδ T cells did not distinguish adults that developed clinical disease or had only asymptomatic infections. It is tempting to speculate that these differences maybe due to altered responses to *Plasmodium* antigens, expression of inhibitory molecules as well as differentiation status of Vδ2 γδ T cells in adults and infants.

## Conclusions

In summary, the results of this study show that semi-immune adults living in an areas of seasonal malaria transmission continue to experience clinical malaria episodes and the heterogeneity in clinical outcomes of *P. falciparum* infections were not dictated by their γδ T cell profiles.

γδ T cells increased during a malaria transmission season and this expansion was noted in both the Vδ2+ and Vδ2− γδ T cells. However, neither expansion or activation of either γδ T cell subsets discriminated study participants that had asymptomatic infections from those that had clinical malaria cases.

## Abbreviations

FMPOS: Faculté de Médecine de Pharmacie et d’OdontoStomatologie
IRB: Institutional Review Board
MRTC: Malaria Research Training Center
NIAID: National Institute of Allergy and Infectious Diseases
RPKM: Reads Per Kilobase of transcript per Million mapped reads
USTTB: University of Sciences, Techniques and Technologies of Bamako
